# Compositional Divergence and Convergence in Local Communities and Spatially Structured Landscapes

**DOI:** 10.1371/journal.pone.0035942

**Published:** 2012-04-26

**Authors:** Tancredi Caruso, Jeff R. Powell, Matthias C. Rillig

**Affiliations:** 1 Institut für Biologie, Plant Ecology, Freie Universität Berlin, Berlin, Germany; 2 Hawkesbury Institute for the Environment, University of Western Sydney, Richmond, New South Wales, Australia; CNRS, University of Montpellier II, France

## Abstract

Community structure depends on both deterministic and stochastic processes. However, patterns of community dissimilarity (e.g. difference in species composition) are difficult to interpret in terms of the relative roles of these processes. Local communities can be more dissimilar (divergence) than, less dissimilar (convergence) than, or as dissimilar as a hypothetical control based on either null or neutral models. However, several mechanisms may result in the same pattern, or act concurrently to generate a pattern, and much research has recently been focusing on unravelling these mechanisms and their relative contributions. Using a simulation approach, we addressed the effect of a complex but realistic spatial structure in the distribution of the niche axis and we analysed patterns of species co-occurrence and beta diversity as measured by dissimilarity indices (e.g. Jaccard index) using either expectations under a null model or neutral dynamics (i.e., based on switching off the niche effect). The strength of niche processes, dispersal, and environmental noise strongly interacted so that niche-driven dynamics may result in local communities that either diverge or converge depending on the combination of these factors. Thus, a fundamental result is that, in real systems, interacting processes of community assembly can be disentangled only by measuring traits such as niche breadth and dispersal. The ability to detect the signal of the niche was also dependent on the spatial resolution of the sampling strategy, which must account for the multiple scale spatial patterns in the niche axis. Notably, some of the patterns we observed correspond to patterns of community dissimilarities previously observed in the field and suggest mechanistic explanations for them or the data required to solve them. Our framework offers a synthesis of the patterns of community dissimilarity produced by the interaction of deterministic and stochastic determinants of community assembly in a spatially explicit and complex context.

## Introduction

Niche theories assume that species competing for a limited set of resources can locally coexist thanks to mechanisms such as life history trade-offs [1]–[3]. Within this framework, species survival is reduced by demographic stochasticity, which sets upper limits to species diversity [3]: as populations become smaller, demographic stochasticity is amplified and the probability of survival is reduced. As long as species possess niches with little overlap, due to narrow niche breadth or relatively large distances between niche optima, niche-based competitive exclusion will be the main process shaping community structure [3]–[5]: all else being equal (e.g. average environmental conditions at a certain scale), these dynamics will lead the local communities of a metacommunity to converge toward a stable composition. However, if the niches of species overlap, their inequality will be strongly reduced and a neutral model may offer a satisfying approximation of processes that mostly shape community structure [4], [6]. From this point of view, limited dispersal actually produces local communities that diverge at increasing geographical distances, with environmental changes playing no role in terms of differences in community structure [7]. Thus, community dynamics resulting from the interaction of dispersal and the niche may produce patterns that vary between matching the expectations of niche theories and having the signature of purely neutral dynamics [8]–[11]. The signature of deterministic processes can be ultimately detected in terms of species co-occurrence patterns: local communities can either converge or diverge in composition with respect to a neutral counterpart or a randomly assembled metacommunity. Instead, earlier tests of neutral theories were based on fitting the zero sum multinomial distribution (ZSM, i.e. the neutral predictions for SAD, i.e. species abundance distribution) to the relative abundance patterns of local communities [12], [13]. However, most evidence suggests that niche and neutral processes may converge in terms of the shape of SAD [8], [14]–[16], which makes the comparison of the performance of different SAD models a weak test (but see [17], [18] for recent theoretical advances). Therefore, it has been suggested that the best approach is to focus on patterns of species co-occurrences and whether these patterns are due to species interaction, stochastic drift and/or habitat heterogeneity [19], [20]. Alternatively, one can use a multitude of approaches, from indices based on taxonomic composition to phylogenetic community structure [11]. Regardless of the specific metric employed to quantify community structure, one approach for analysing data from field studies is to generate appropriate null models [19], [21] from the data matrix structure. This method is effective when the sampling design accounts for the spatial scales [22], [23] at which the differential role of neutral and niche dynamics leaves distinguishable signatures [20], [23], [24]. Along a steep gradient, and under strong niche dynamics, species should tend to co-occur less often than expected by chance (segregation; e.g. [19]). The proponents of null models also argue that in real observational data one cannot switch niche dynamics off. Thereby, in the specific case of the niche-neutral debate, one needs to create null matrices based on randomisation schemes that nullify the patterns of species co-occurrence expected under species interaction (e.g., competitive interaction; e.g. algorithm SIM 9 in Gotelli [19]).

Several authors have recently proposed that neutral models offer a more mechanistic approach for generating expectations for patterns of community structure [17], [25]–[28]. For instance, by estimating the neutral diversity (*θ*) and immigration (*m*) parameters from field data (e.g. [17], [28]), hypothetical data sets can be simulated to estimate patterns of community dissimilarity and beta-diversity expected under neutrality. The above procedures create a neutral (i.e., based on population dynamics) expectation that can be compared to the observed data [17], [25]. In fact, some authors proposed that null models based on randomisation of real data [19], [29] may generate unrealistic expectations because they do not account for stochastic population dynamics [27]. The rationale behind this idea is that classical null models based on randomising real data do not really embody population dynamics.

Recent analyses based on simulations [4], [5], [11], [30], field data [20], [25], [31]–[33], and employing either null, neutral or both null and neutral models have successfully addressed the relative roles of stochastic and deterministic processes and distinguished their different signatures in terms of levels of community dissimilarities.

However, we are not aware of simulation studies that explored the effects of three key components: i) an explicit and complex spatial structure in the niche axis that involves non-linear, periodic terms and random noise [22]; ii) an assessment of the ability of null models to detect non-random patterns under conditions that are close to ecological neutrality (e.g., broad niche); and iii) a comparison of sampling strategies that differ in terms of grain (the size of sampling quadrat) and resolution (spatial frequency of sampling quadrats, which determines the power to detect non-linear periodic structures; [22], [34], [35]). These three factors are important for the following reasons: i) on a certain spatial scale, average levels of community dissimilarity, as measured by indices such as the Jaccard index [36], depend on spatially explicit dynamics in terms of both dispersal and the distribution of the niche component [11]; ii) null models based on conservative randomization schemes (low rate of Type I error; e.g. SIM 9 in Gotelli [19]) and classical metrics such as the C score [19], [29] might not detect the signature of deterministic processes that are actually taking place; iii) observed patterns will depend on the spatial scales accounted for by the sampling design. However, when the environment is characterised by complex spatial structures, it is likely that real sampling designs can address only some of the spatial scales at which community dynamics take place [34]. This can lead to the failure of null models.

**Figure 1:**
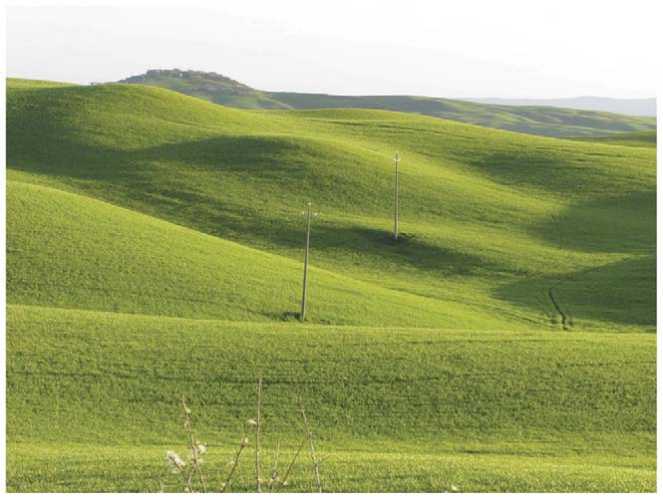
Typical Tuscan countryside near Siena (Italy). The pictured landscape is known as “Le Crete” and is characterised by gentle hills and slopes. This photo demonstrates the concept of an environment with multiple spatial structures (*sensu* Borcard et al. 2004). In the picture, one can clearly see a linear trend corresponding to the average slope of the terrain but also a sinusoidal pattern in the way hills and valleys alternate along the linear gradient. We used this view for simulating the continuum hypothesis in a spatially structured landscape. Credit: Giuseppe Manganelli, University of Siena.

**Figure 2:**
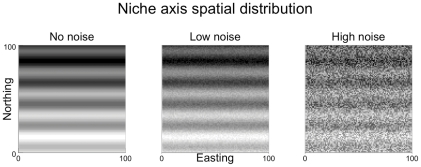
Spatial distribution of the niche axis, which is the parameter determining propagule survival (see addition methods in the supporting information for a quantitative description). From white to black, the niche *E* ranges from value one to 100. On the left panel, the systematic component in the spatial distribution of *E* is shown: a linear trend makes the niche having light tones in the south and progressively darker tones toward the north; further, a periodic component was added, that generates a sinusoidal-like patterns (compare to Fig. 1). Panels in the middle and the right sides show the effect of adding respectively low (range of uniform distribution = 10) and high noise (range = 100) to the pattern on the left side. Even when the noise is high (right side), some spatial pattern is still visible.

Here we use a heuristic simulation framework, which takes into account all these factors. We explored patterns of community structure that are expected in spatially structured environments (Figure 1 and 2) by virtue of different combinations of niche breath, dispersal limitation, and environmental stochasticity, finding that these features interact in determining variable levels of community dissimilarity. Indeed, neutral or null models are useful to detect the signature of deterministic processes in terms of local communities that diverge or converge with respect to their neutral or null counterpart but there can be many mechanisms behind this signature. The present study sheds light on these mechanisms and offers a framework for synthesising the patterns of dissimilarity they produce.

## Methods

### Model

We based our simulation procedures on the approaches developed by Tilman [3] and Gravel and co-workers [4] for formulating the continuum hypothesis: purely niche and purely neutral dynamics actually are the two extremes of a continuum. Between those extremes, niche and neutral processes can act concurrently.

We implemented our simulation model in R [37] using the package “simecol” [38] and in Data S1 we provide our original code, which includes detailed technical information. The theoretical basis of our work [3], is briefly recalled in Text S1 as well. In summary, we generated individual-based models describing the recruitment dynamics as a lottery process [7]. Basically, we created a grid of 10,000 cells in a spatially explicit environment with both the northing and easting coordinates ranging from the dimensionless value 1 to 100. Each cell represents an adult picked from a pool of 20 species. At the beginning of each simulation, individuals were randomly drawn from the species pool in order to create random starting assemblages. Population dynamics were: 1, adult stochastic mortality; 2, dispersal; 3, juvenile survival dependent on the stochastic, Gaussian niche (see also eq. 1 and 2 in Text S1; [4]), which ultimately creates a pool of seedlings for each cell and from which to randomly recruit in order to replace the dead adult. Thus, the probability of a species to conquer a cell depends on its relative abundance in the pool of seedlings and this relative abundance depends on two components: the niche and dispersal limitations. By varying niche breadth and mean dispersal distance (i.e., the kernel's variance), it is possible to create scenarios that differ in their degree of “neutrality” or, conversely, of niche partitioning. Differently than the authors from whom we took inspiration [4], spatially explicit dispersal was modelled using a bivariate Gaussian kernel. In fact, as recently demonstrated by Chisholm and Lichstein [18], the shape of the kernel is not relevant to the aim of our analysis since it does not affect the analytical relationship that links immigration rates to the mean dispersal distance *d*. Given the malleability of the Gaussian, we preferred it for mathematical convenience. Under neutrality, any community in any closed landscape would converge on one species [7] if there were not a metacommunity speciation term or an external immigration flux maintaining diversity. Following Gravel and co-workers [4], we introduced implicit immigration, which contributed to about 10% of the propagules immigrating to each cell [7], [39]. That is to say that within each cell 90% of the propagules came from immigration internal (i.e., spatially explicit) to the modelled landscape while 10% were randomly sampled from a uniform distribution (spatially implicit immigration). This implicit immigration depends, for example, on fecundity, which was assumed to be equal among species. Fecundity contributes to determining the mean percent contribution of “external” immigrants to the pool of seedlings within each cell [4]. This is the pool from which it is necessary to recruit after a cell occupant happened to die by stochastic mortality (mortality rate was set to 25%). In principle, the results from any neutral model are also sensitive to the implicit immigration rate and the distribution of species in the source pool. However, preliminary results we used for calibrating these model parameters (i.e., external and implicit immigration, and mortality) confirmed that results relevant to our aims are not sensitive to our choice of fecundity and mortality rates or the shape of the metacommunity SAD from which spatially implicit immigration was generated.

To model the “niche filter”, some authors have used an environmental grid with a mesh arbitrarily large and with each cell having a value randomly drawn from a uniform distribution, which ranged from 0 to 100 [4]. Then, they modelled the system as a torus in order to avoid edge effects. Here, we assume that natural communities are spatially constrained (e.g., soil communities in a patch of a forest surrounded by grasslands or tropical coral reefs surrounded by sandy substrates) and within these spatial constraints experience gradients in the spatial distributions of resources and conditions [2], [22], [35]. In order to introduce this spatial component, we associated a niche value to each individual cell using three features ([11], [22]; Fig. 2): 1, a linear trend from the “south” to the “north”; 2, a periodic component that was modelled by a sinusoidal function; 3, a random component drawn from a uniform distribution. The random component represents natural noise due to local irregular variation in the spatial distribution of the niche. We also created landscapes consisting of just the random component and compared them with the spatially structured landscape in order to ensure that observed community patterns actually depended on the spatial structure. This was indeed the case (see Table S1) and we therefore report and discuss in the main text only the results based on the 18 simulation scenarios that were based on the parameters given below. Our aim was to reproduce natural, virtually continuous spatial structure such as the one pictured in Figure 1. Classically, noise has been modelled in terms of temporal stochastic fluctuations [5]. However, it can also be introduced in terms of stochastic spatial fluctuation of resources or conditions [22], [40]. The two could also be combined but given our aim we maintained a fixed environment through the time steps of our simulated dynamics. The niche optima of the 20 species were regularly spaced along the niche axis range.

### Simulation scenarios

We created 18 scenarios accounting for different combinations of three factors, which respectively consisted of different levels of niche breadths, dispersal and environmental noise. Niche breadth consisted of three levels: narrow (σ_n_ = 0.5), medium (σ_n_ = 25), and broad (σ_n_ = 50). Dispersal consisted of three levels as well: low (σ_k_ = 5), intermediate (σ_k_ = 45), and high (σ_k_ = 85). Noise consisted of two levels: low (range of uniform distribution = 10) and high (range = 100). We stress that the noise level “high” did not nullify small-scale patterns (as evident in Figure 2). Each scenario was simulated for 5000 time steps, which allowed communities to reach stability in terms of species richness and turnover (see also [34] and examples given in Figure S1).

### Sampling and data analysis

We used two different sampling approaches: one using a plot size (grid *sensu* [34]) that matches the periodic structure in the environment (fine resolution), and another using a larger plot size (coarse resolution, Figure S2). Both approaches used the same sampling effort in terms of total sampling area. Thus, the reason why the sampling scheme with finer grid has more resolution is that it consists of smaller but more numerous sampling plots. This implies that the average interval separating the sampling units is (within a latitudinal stratum, see below) shorter than that of the coarse sampling strategy. Thereby, this fine strategy captures the spatial periodicity in the niche axis at the right scale. Also, this sampling strategy is based on a definition of local community that, in terms of size, better suits the scale at which assembly processes are operating. These differences in sampling strategy are realistic and account for the fact that in reality it can be difficult to decide the right scale of the sampling strategy in terms of grid, interval and overall extent [34]. Thus, the rationale for comparing these sampling approaches is that we were interested in comparing “true” patterns of community convergence/divergence to those that arise from a mismatch to the structure of the environment at a fine scale (as may often occur in the field). Samples were stratified latitudinally, dividing the landscape into thirds (excluding the ten cells on the edge of the landscape) and focusing on the two strata to the north and the south, and longitudinally. Each sampling procedure was replicated six times for each of the landscapes generated by our 18 simulation schemes and analyses were performed at two spatial scales: between the two latitudinal strata and within a latitudinal stratum. Sampling the same landscape multiple times greatly reduced computational times. We could adopt this strategy since preliminary simulations demonstrated that replicating the sampling within a landscape gave results comparable (no significant differences on average) to results obtained by replicating the entire landscape and then sampling each and every landscape only once (see example given in Table S2 and S3).

For each sample, we conducted a null model analysis [19], [21]. There are many possible options for running null model analyses, depending on the hypothesis under investigation. In the framework of the continuum hypothesis [4], the most relevant approach is to focus on the effect of species co-occurrence patterns alone. This is fairly easily achieved by fixing row and column sums during the randomisation process (algorithm SIM9 in Gotelli [19]). This procedure constrains randomised matrices very much in terms of number of species per site (which is fixed) and the number of sites where a certain species may occur (which is fixed as well). However, the latter constraint is the fundamental one when testing for co-occurrence patterns [19]. Thus we also tried to constrain our randomisation scheme by only fixing rows (species; SIM2 in [19]. In practice, we observed no differences between the output from the two algorithms and we therefore present results from the algorithm SIM9 [20], [23]). We performed 5000 randomisations in order to create null expectations for the C-score, an index of species co-occurrence based on measuring checkerboard patterns [19], [23]. In our case, the null model generates random patterns expected under no species interaction, which in our scenario is indirectly accounted for by the effect of the niche axis. However, the advantage of simulation approaches is that one can really switch off the mechanisms that are known to create community patterns. By nullifying species niche differences we were able to create a neutral expectation of community patterns. In order to do so, we ran a series of simulations creating strictly neutral equivalents of the 18 simulation scenarios by equalising niche optima (at the centre of the environmental niche axis) and breadth (using σ_n_ = 25), resulting in species distributions being determined entirely by dispersal and demographic drift. Each of the 18 scenarios was thus compared to its neutral counterpart generated using the corresponding dispersal range. We quantified the difference between the “real” communities and their neutral counterparts by calculating the dissimilarities (measured by Jaccard's index) between each pair of samples collected across the simulated landscapes [36]. Results were not influenced by the choice of the dissimilarity coefficient (data not shown; we tested Bray-Curtis, Gower and Sørensen). We thus created one distribution of dissimilarities for the “real” community and one for the neutral communities. The differences between the two distributions were quantified using t-statistics. We note that this simulation approach has its real counterpart in model-based neutral approaches applied to real data [17], [25], [28]. Of course, in the case of real data sets one has to estimate neutral parameters and use these estimates to generate a neutral expectation while in the case of simulations the neutral expectation is simply based on those spatially explicit scenarios that have been generated by neutral dynamics (i.e., the niche mechanism is known and can be switched off). Thus, given our aims, there was no need to estimate neutral parameters using the model-based approach. Actually, this would have been misleading because current neutral models that allow testing of their fit to data are limited in that they are spatially implicit [41] while we had to create spatially explicit neutral scenarios.

We used linear models (ANOVA full factorial design) to estimate the effects of sampling design and the analytical approach (null *vs* neutral) on the extent to which the signal of niche-based processes could be detected, including niche breadth (three levels), dispersal (three levels), noise (two levels), and all two-way interactions as factors in the model. In essence, our approach is analogous to a meta-analysis [42] in which we used standardised effect sizes to estimate responses (i.e. standardised C-score for null models, t-statistic for the neutral approach), both of which are expressions of the distance from the mean null expectation relative to the variance in the distribution of null expectations. In practice, in the case of the C-score, we calculated the difference between observed and expected (i.e. the random matrices) C-score and divided it by the standard deviation of the expected matrices. In the case of the neutral analysis, we simply used the definition of the t-statistic (which in practice is an effect size expressed in units of standard deviations). Data were normally distributed. We decided to focus on these original statistics and not on developing new approaches (e.g., dissimilarities expected from statistical null models) since the statistics we analyzed are the ones commonly employed in the relevant literature. Importantly, these statistics basically convey the same qualitative idea: local communities may either diverge/segregate or converge/aggregate with respect to their neutral/null counterpart.

## Results

**Figure 3:**
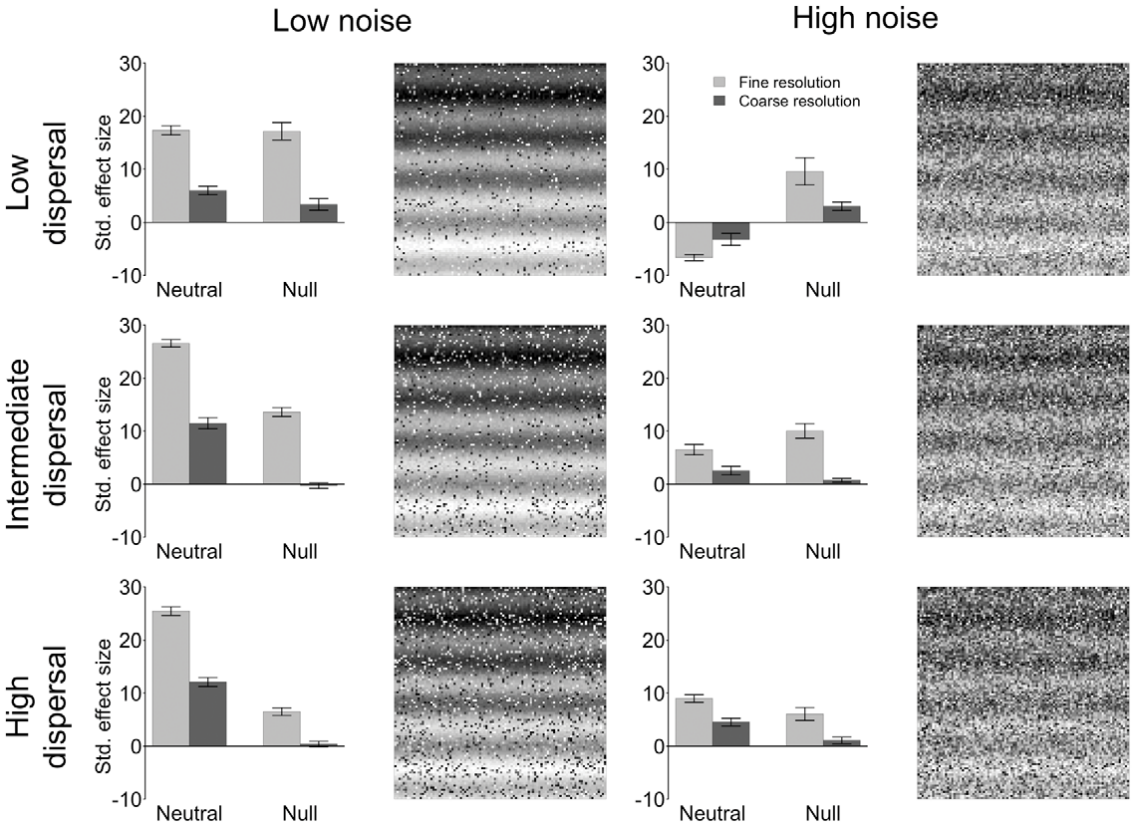
Six of the simulated communities after 5000 time steps. Beside each simulated landscape, mean (± S.E.) standardised effect size are reported with data stratified by type of null hypothesis (neutral *vs.* null) and sampling design. For the neutral analysis, a positive effect size means that local communities under the effect of the niche are more dissimilar than their neutral counterpart (i.e., niche switched off by nullifying niche differences). For the null model, a positive effect size means that species are co-occurring less than expected by chance (segregation). Here we present the results for narrow niche breadth stratified by dispersal (rows) and noise (columns). In the top left corner, low levels of dispersal and noise produce clearly visible spatial patterns that become more confused (see also Fig. 4 and Fig. 5) as noise and dispersal are increased. In the bottom right corner, parameter settings are opposite to the top left corner and species distributions appear highly stochastic, even though a careful visual examination of the gray tones reveals some perceivable spatial patterns in terms of the periodic component. Results are reported for the analysis performed between the two latitudinal strata (north and south). The results for the analysis performed within a latitudinal stratum are reported in the supplementary material and reinforce patterns visible in this figure and Fig. 4 and 5.

**Figure 4:**
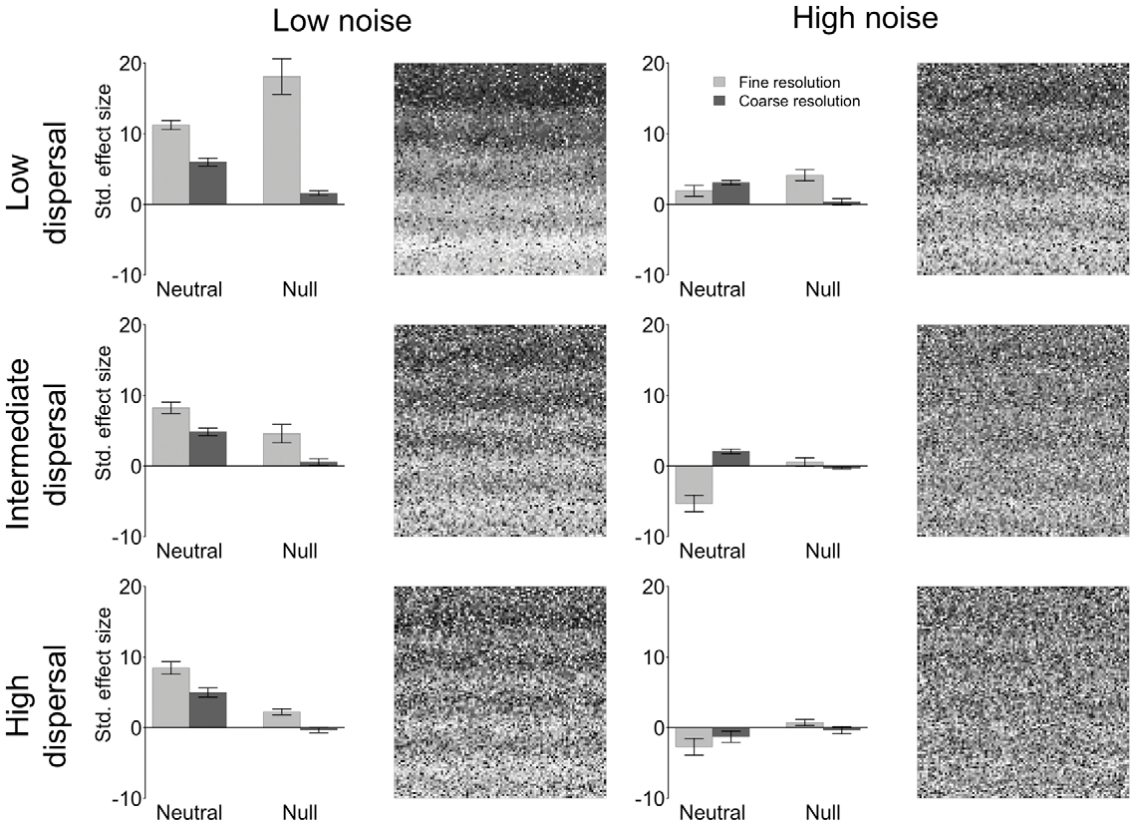
As **Fig. 3** but for the intermediate niche breadth.

**Figure 5:**
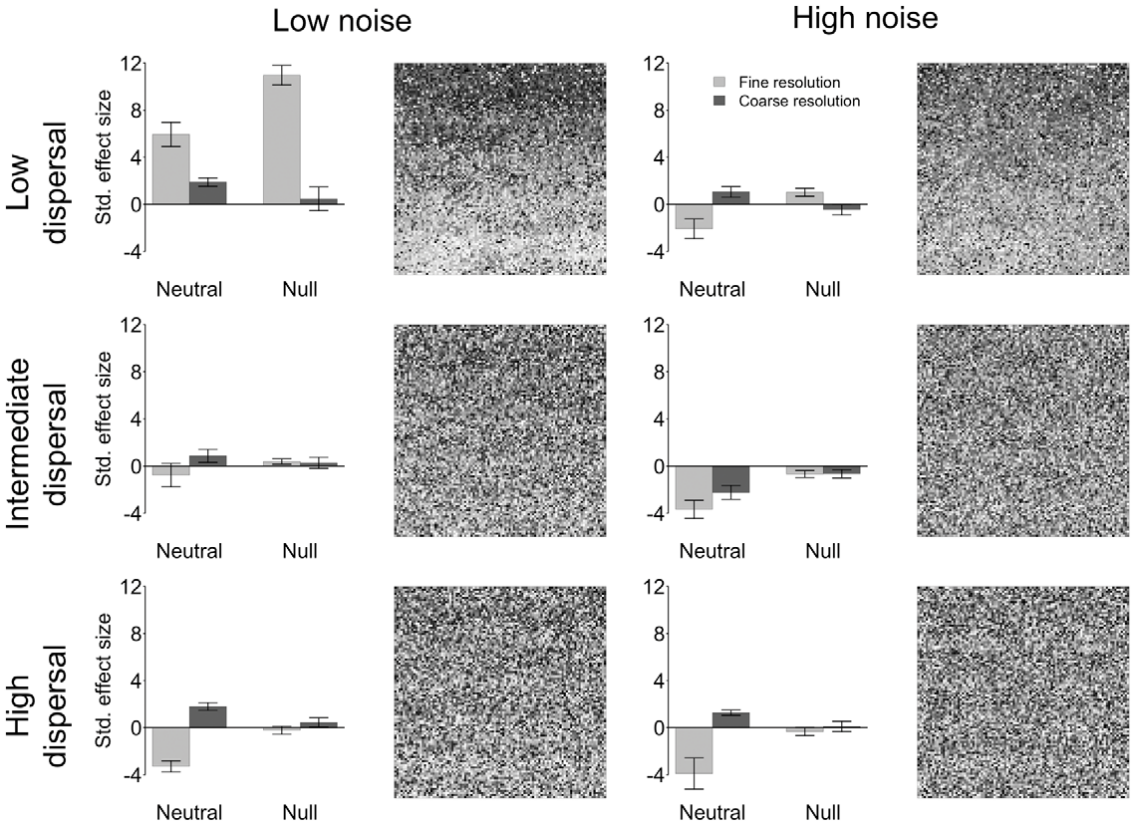
As **Fig. 3** but for the broad niche breadth.

The 18 simulation scenarios generated by our approach produced visual patterns representing varying degrees of spatial structure in species distributions (Figure 3, 4, 5, Figure S3, S4, S5). On the one hand, species distributions (see simulated landscapes in Figure 3, 4, 5, Figure S3, S4, S5) clearly follow the distribution of the niche axes (Figure 2) in scenarios based on narrow niche breadth (Figure 3). On the other hand, species distributions are much less spatially structured in scenarios based on broad niche breadth (Figure 5). Intermediate levels of spatial structuring are observed at intermediate niche breadth (Figure 4). Niche breadth being equal, increasing dispersal distance makes species distributions less structured (e.g., Figure 5, compare the three levels of dispersal). At high noise, spatial structures are less visible, even when niche breath is narrow and dispersal low (compare the two top panels of Figure 3). At the very extreme combination of high noise, broad niche breadth and high dispersal, there is little visible spatial structure in species distributions (bottom right corner of Figure 5). Regarding this specific scenario, it is instructive to compare it with Figure 2, high noise panel, which shows the distribution of the niche axis that underlies the dynamics of this specific scenario. This comparison emphasizes how random species distributions can be despite the deterministic effect of the niche axis, which still contributes to species population dynamics. Indeed, the high unpredictability of species distributions results from the high levels of stochasticity introduced by high variance in niche breadth and dispersal distance.

Spatial patterns at the scale of the landscape were generally detected (see bar plots next to the simulated landscape in Figure 3, 4, 5 and Figure S3, S4, S5) by both null models (bars labelled “Null”) based on randomisation of sampled data (see Figure S2 for the sampling strategies) and neutral scenarios (Figure 3, 4, 5, Figure S3, S4, S5), bars labelled “Neutral”), which were obtained by switching off the niche component (see methods for details). Indeed (Table 1 and Table S4), niche breadth (*P*<0.001), dispersal distance (*P*<0.001), and environmental noise (*P*<0.001) were generally detected as factors significantly affecting species co-occurrence (C-score, null model analysis based on algorithm SIM 9 in Gotelli [19]) and beta diversity as measured by the Jaccard index (i.e., neutral analysis comparing dissimilarity in species composition between niche based scenarios and their neutral counterpart). Generally, for both approaches, effect sizes were larger for more spatially structured species distributions (e.g., compare top panels of Figure 3 and 5). However, the most interesting pattern is that interactions among the three simulation factors (niche, dispersal, noise), the employed methods (sampling *vs.* analytical strategy), and these two types of factors (simulation factors *vs* methods) were also observed (Table 1 and Table S4).

**Table 1:** ANOVA table for the linear model with Standardised Effect Size (**between** Latitudinal zones; see methods for details) as the response variable.

Effect	Df	Sum Sq	Mean Sq	F value	Pr(>F)
Method (Neutral vs Null)	1	106	106	8	<0.001
Resolution (Fine vs Coarse)	1	1388	1388	95	<0.001
Niche Breadth (Narrow, Medium, Broad)	2	3892	1946	133	<0.001
Disperal (Low, Intermediate, High)	2	216	108	7	<0.001
Noise (Low vs High)	1	3114	3114	213	<0.001
Method:Niche Breadth	2	379	190	13	<0.001
Method:Resolution	1	301	301	21	<0.001
Resolution:Niche Breadth	2	1328	664	45	<0.001
Niche Breadth:Dispersal	4	872	218	15	<0.001
Resolution:Noise	1	888	888	61	<0.001
Method:Noise	1	883	883	60	<0.001
Residuals	413	6034	15		

Factors were Method of analysis (neutral, null), Sampling design (fine, coarse), Niche Breadth (narrow, medium, broad), Dispersal (low, intermediate, high) and noise (low and high). This table shows overall main and interaction (:) effects.

Df, degrees of freedom; Sum Sq, sum of squares; Mean Sq, mean sum of squares.

Here, we highlight those interactions that are most relevant to the aim of our study. Firstly, a mismatch of the plot size to the fine-scale structure of the environment resulted in a reduced ability to detect the “true” pattern of community divergence/convergence (Figure 3, 4, 5). In fact, the coarse sampling strategy based on relatively large plots was, in some instances, not able to detect the effect of the periodic component in the niche axis. In scenarios with low niche overlap and low environmental noise, this “error” was simply represented by a reduced effect size (Figure 3) and the two sampling strategies were still consistent in terms of the direction of the effect, which in this case generally was positive (that is to say divergence for the neutral approach and species segregation for the null model approach). However, in some scenarios resulting in convergence between local communities (e.g., Figure 5, bottom panels, neutral approach, light grey bar) a mismatch between plot size and environmental structure (coarse sampling design) resulted in an erroneous signal consistent with divergence (Figure 5, bottom panels, neutral approach, dark grey bar). The large plot size implied sampling at a scale coarser than the process under investigation (spatial patterns at fine scale averaged over a coarser scale) and caused local communities to seem more dissimilar with respect to their neutral counterpart. Finally, in several cases coupling the coarse sampling strategy with null model analysis resulted in no signal, even when spatial structure was clearly visible (e.g., Figure 5, top left corner and null, dark grey bar).

Finally, as niche breadth, dispersal and noise increased, both the null and neutral approaches lost power (small and in some case no significant effect size) also when spatial patterns were clearly visible at the scale of the entire landscape (e.g., Figure 5, intermediate and bottom panels). However, there were instances (e.g. Figure 5 bottom panels) where the null model detected no signal while the neutral approach did.

## Discussion

### Neutral approach: revealing mechanisms underlying community dissimilarity

Communities driven by a mixture of stochastic (neutral dynamics) and deterministic (niche partitioning) dynamics produce patterns that show the signature of the niche when there is explicit structure in its spatial distribution, even when they can be partially approximated by the dynamics assumed by neutral models [5], [43]. As expected, in our models, niche processes caused communities to be on average significantly more or less dissimilar than their neutral counterpart at scales where the niche axis varied enough to dominate population dynamics. On the one hand, communities tended to diverge (more dissimilar than their neutral counterpart) whenever spatial structures in species distributions were clearly driven by the spatial structure of the niche axis (e.g. narrow niche breadth and low dispersal, but see how high noise might reverse the pattern: Figure 3, compare top right panel with the top left one). On the other hand, communities tended to converge at broad niche breadth and intermediate to high dispersal, which in practice make the environmental filter less rigid by allowing species to colonise environments that are relatively far from species' theoretical optima. This resulted in less visible spatial structures in species distribution.

It is generally thought that neutral processes lead on average to spatial divergence between communities since community dissimilarity is determined by the strength of dispersal limitation [7], [24], [44]–[46]. On the other hand, environmental conditions being more or less constant across a certain scale, niche partitioning is generally thought to lead to higher levels of community similarity [19], [20], [24], [47] because the assemblage deterministically converges towards some equilibrium species composition. Our data clearly show that, sampling strategy being equal, the real mechanisms behind convergence and divergence actually depend on a balance among dispersal, niche breadth and environmental noise. For example, population dynamics being equal in terms of dispersal and niche, communities may either diverge or converge depending on whether noise is low or high, respectively (Figure 3: compare top panels, neutral bars).

The direction of the effect (positive for divergence, negative for convergence) also depends on the magnitude of dispersal and the extent of niche overlap, indeed on their interaction (Figure 3, 4, 5; Table 1). This result thus offers new interpretation for patterns previously observed in the field. For instance, in an empirical example of coral communities, Dornelas and co-workers [25] observed mean level of community dissimilarity values and their variances that were much higher (divergence) than the neutral theory predicted and interpreted these results as an effect of spatio-temporal environmental stochasticity driving patterns of diversity in coral reefs. In fact, a disturbance regime may push local communities toward unique composition by continuously resetting colonisation processes. In our simulations, there were basically two conditions analogous to spatial disturbance (high noise scenarios) that produced divergence: either narrow niche breadth was coupled with intermediate or high dispersal or broad niche breadth was coupled with low dispersal. Therefore one or a mix of these two mechanisms may have been at work in the communities under study by Dornelas and co-workers [25]. Thus, our models suggest that data on the extent of niche overlap and dispersal limitation are necessary to disentangle these two mechanisms and assess their relative effects. This will allow going from patterns indicating the signature of deterministic processes (divergence or convergence) to the mechanisms behind these patterns.

### Null models and the effect of sampling strategy

In real systems, one may create a stochastic expectation of community structure by either fitting a neutral model [17], [25], [28], [32], [33] or randomising data matrices following the logic of null models [19]. Note that fitting a neutral model, estimating neutral parameters and generating a null expectation for observed data is not what we have done in this study since, in our case, we were able to create truly neutral landscapes and spatially explicit expectations under neutral dynamics [30]. In the real world, fitting spatially explicit neutral models still is a research frontier [41]. As regards null model approaches, hypotheses relevant to the relative roles of stochastic and deterministic processes of community structure have usually been framed in terms of species co-occurrence patterns [20] though several metrics can actually be employed [11]. An interesting point is to address under which conditions metrics accounting for species co-occurrence patterns are able to detect the signature of deterministic components of community assembly, which in our case were represented by a spatially structured niche axis. Our results show that non-random patterns are detected only where the resolution of the sampling design accounted for broad and fine scale patterns in terms of plot size and the interval separating plots [22], [34], [35], [48]. In fact, this is the only way to let the sampling design match not only the linear gradient but also the periodic component in the spatial distribution of the niche. This result is also consistent with theoretical predictions obtained by recent simulation studies [30] and has been experimentally validated in terms of null model approaches by several authors [20], [23]. In addition to that, in environments that have complex spatial structures, the ability to detect signals using null model analysis can be constrained to a specific set of circumstances: combining high noise with high dispersal and broad niche breadth made null models much less powerful. However, null model analysis of species co-occurrence was generally able to detect the signature of deterministic processes, especially when the sampling design matched the scale of the process under investigation (fine resolution sampling regime).

In the few cases where null models failed to detect non-random patterns, the neutral analysis shows that community assembly was actually affected by the deterministic component due to the niche. This emphasises that even when the sampling design is conceived at the right spatial scale, null model analysis (at least in terms of our set up: see methods) might simply not have enough power to detect non-stochastic processes. This certainly applies to environments characterised by high noise as well as to population dynamics close to neutrality in terms of niche breadth (broad).

### Perspectives and conclusions

While we attempted to incorporate a wide range of scenarios into our simulation framework, certain limitations may prevent extrapolation of these results to different scenarios. In particular, our results are of special relevance to environments that are known to have clear spatial gradients and some periodicity in the distribution of the co-varying variables that are hypothesised to be the major drivers of species distributions. These features are certainly common, for example, in terrestrial habitats such as the soil (Fig. 1; [49]). Our interpretation is valid for community patterns that depend on spatial structures analogous to the one we analysed in our simulation. Even though this structure is general enough to allow a first heuristic approach [22], [35], other spatial patterns are possible and should likewise be explored [11], [30]. For example, we explored the effect of one niche axis but our simulation schemes can be easily extended to a multivariate system of niche axes that could correspond to different species traits and with variable spatial patterns. Also, we analysed niche effect in terms of environmental filtering and we did not introduce competition terms in an explicit way, which is perhaps more relevant to the general debate around neutral theories [7], [50]. Furthermore, we basically analysed local dynamics and assumed an implicit, minimal source-sink mechanism necessary to maintain the diversity of our landscape under a neutral scenario but in nature meta-communities are much larger than the local community, and our heuristic analysis should be extended to include the explicit dynamics that in real landscapes link local and metacommunities.

Despite these limitations, our results clearly showed that the occurrence and detection of highly similar or divergent local communities depended on interactions between niche breadth, dispersal and environmental noise, which are all ecological features of the community, and sampling design, which is a general methodological aspect that addresses specific biological features (e.g., the scale of dispersal). Indeed, the direction of the effect we observed was dependent on the specific combination of dispersal rate and the extent of niche overlap, which demonstrates the importance of focusing more on the relative roles of these two processes than on whether one or the other determines community structure. In particular, a key conclusion is that under certain conditions processes of community assembly can be disentangled only by measuring traits such as niche breadth and dispersal. Our framework thus offers a synthesis of the patterns of community dissimilarity produced by the interaction of deterministic and stochastic determinants of community assembly.

## Supporting Information

Figure S1 **Temporal dynamics in simulated communities generated under the scenario of low dispersal, narrow niche breadth, and low environmental noise.** Local communities were sampled using the “coarse resolution” sampling design (see methods). The figures clearly show that an equilibrium level of alpha- and beta-diversity (points = mean, bars = standard error) was reached well before the communities were sampled for the analyses reported in this paper (after 5000 generations). Equilibria were also observed for other scenarios, although at differing levels of alpha- and beta-diversity. For clarity of representation time points are shown that follow a geometric progression.
(TIF)

Figure S2 **The two sampling strategies: fine resolution with smaller plots on the left and coarse resolution with larger plots on the right.** Both strategies can detect the main environmental gradient potentially affecting community structure and running from the south to the north (Figure 2). However, the fine resolution strategy, total surveyed area being equal, consists of smaller (five by five instead of ten by ten) but more densely distributed plots, which allows to solve fine spatial patterns, in particular the periodic component we introduced in the distribution of the niche axis. Basically, the two sampling designs are based on stratifying by latitude (north and south stratum), with plots replicated longitudinally. The longitudinal replication is spatially randomized and may lead to different outcomes in terms of the exact position of each plot.
(TIF)

Figure S3 **Results of the analysis performed within one of the latitudinal strata for narrow niche breadth stratified by dispersal (rows) and noise (columns).** Next to each simulated community, mean (S.E.) standardised effect size are reported with data stratified by type of null hypothesis (neutral vs. null) and sampling design.
(TIF)

Figure S4 **Results of the analysis performed within one of the latitudinal strata for intermediate niche breadth stratified by dispersal (rows) and noise (columns).** Next to each simulated community, mean (S.E.) standardised effect size are reported with data stratified by type of null hypothesis (neutral vs. null) and sampling design.
(TIF)

Figure S5 **Results of the analysis performed within one of the latitudinal strata for wide niche breadth stratified by dispersal (rows) and noise (columns).** Next to each simulated community, mean (S.E.) standardised effect size are reported with data stratified by type of null hypothesis (neutral vs. null) and sampling design.
(TIF)

Table S1 Comparison of scenarios based on random spatial distribution with scenarios based on a spatially structured niche axis. Values refer to average standardised effect size (mean ± S.E.) of the C-score from null model analysis.
(DOC)

Table S2 Comparing results from multiple sampling within one landscape with results from sampling replicated landscapes. Values refer to average standardised effect size (mean ± S.E.) of the C-score from null model analysis.
(DOC)

Table S3 Comparing results from multiple sampling within one landscape with results from sampling replicated landscapes. Values refer to average standardised effect size (mean ± S.E.) from neutral analysis.
(DOC)

Table S4 ANOVA table for the linear model with Standardised Effect Size (**within** Latitudinal zones; see methods for details) as response. Factors were Method of analysis (neutral, null), Sampling design (Fine, Coarse), Niche Breadth (narrow, medium, broad), Dispersal (low, intermediate, high) and noise (low and high). This table shows overall main and interaction (:) effects.
(DOC)

Text S1 The theoretical basis of the work is briefly recalled.
(DOC)

Data S1 The original R code used to simulate community dynamics.
(R) Click here for additional data file.
