# Supplementary figures and images for: Compositional Divergence and Convergence in Local Communities and Spatially Structured Landscapes
Source: PLoS One. 2012 Apr 26;7(4):e35942. doi: 10.1371/journal.pone.0035942 (PMC3338555; doi:10.1371/journal.pone.0035942)

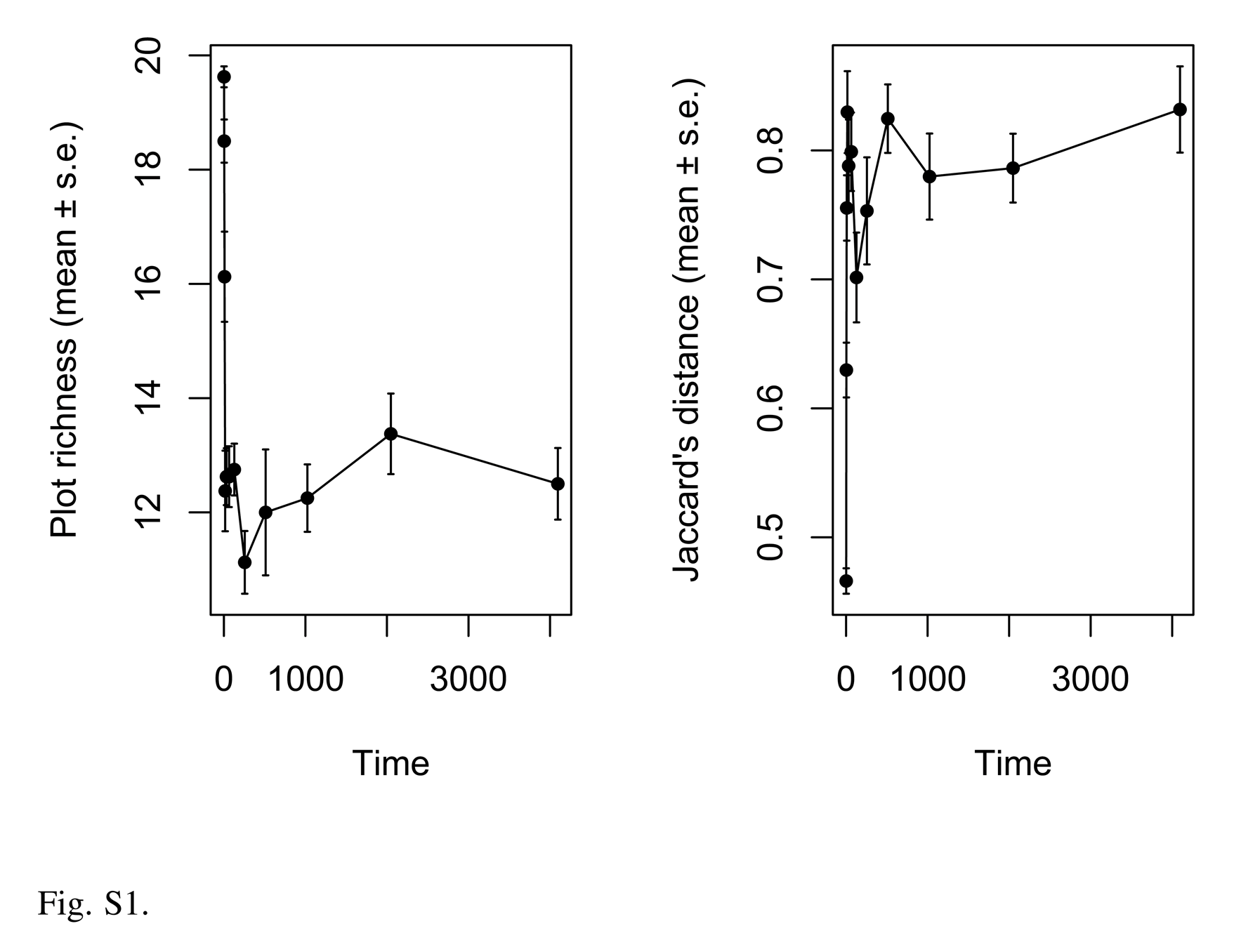

Supplement: Figure S1 — Temporal dynamics in simulated communities generated under the scenario of low dispersal, narrow niche breadth, and low environmental noise. Local communities were sampled using the “coarse resolution” sampling design (see methods). The figures clearly show that an equilibrium level of alpha- and beta-diversity (points = mean, bars = standard error) was reached well before the communities were sampled for the analyses reported in this paper (after 5000 generations). Equilibria were also observed for other scenarios, although at differing levels of alpha- and beta-diversity. For clarity of representation time points are shown that follow a geometric progression. (TIF) [file pone.0035942.s001.tif]

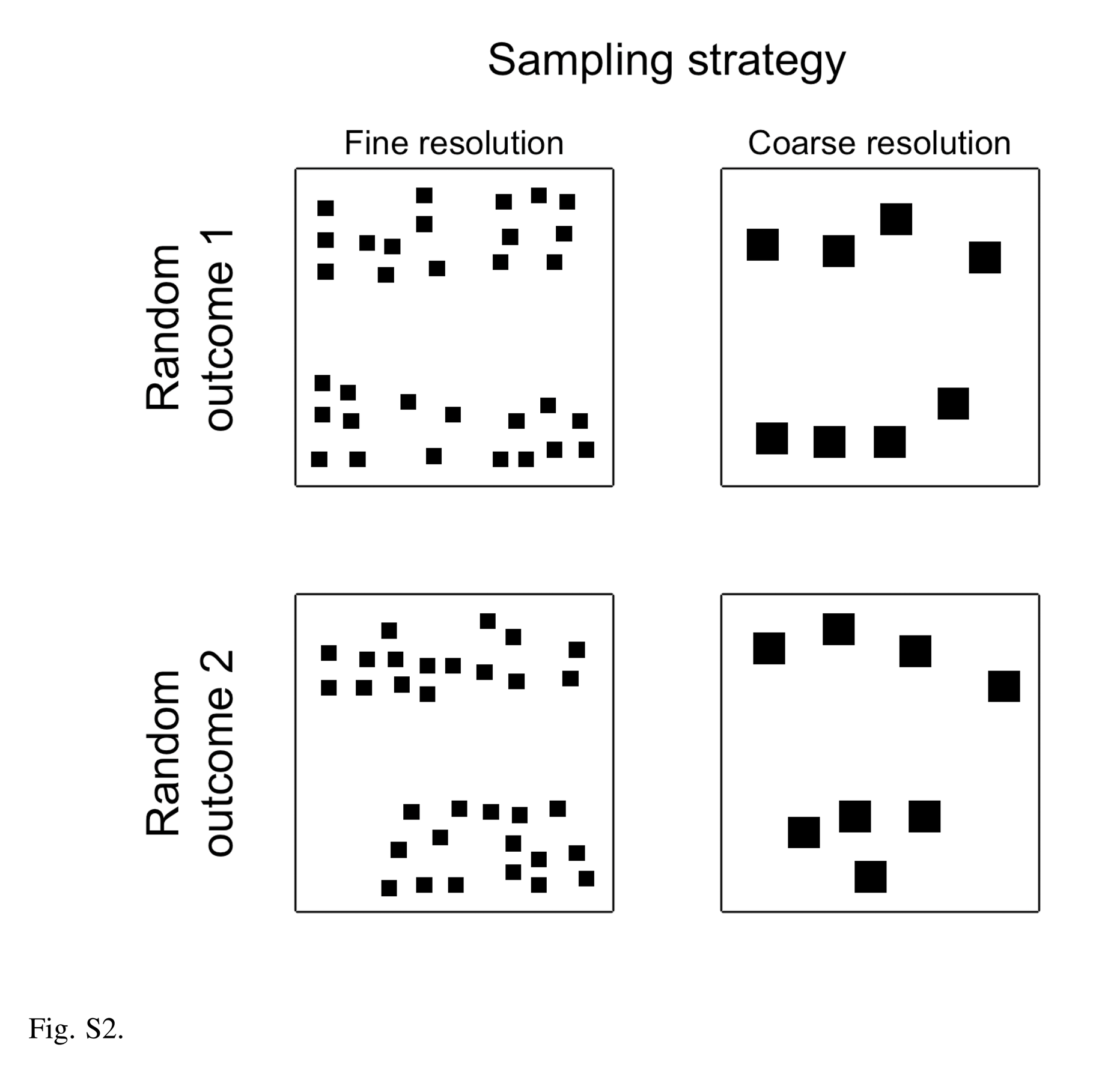

Supplement: Figure S2 — The two sampling strategies: fine resolution with smaller plots on the left and coarse resolution with larger plots on the right. Both strategies can detect the main environmental gradient potentially affecting community structure and running from the south to the north (Figure 2). However, the fine resolution strategy, total surveyed area being equal, consists of smaller (five by five instead of ten by ten) but more densely distributed plots, which allows to solve fine spatial patterns, in particular the periodic component we introduced in the distribution of the niche axis. Basically, the two sampling designs are based on stratifying by latitude (north and south stratum), with plots replicated longitudinally. The longitudinal replication is spatially randomized and may lead to different outcomes in terms of the exact position of each plot. (TIF) [file pone.0035942.s002.tif]

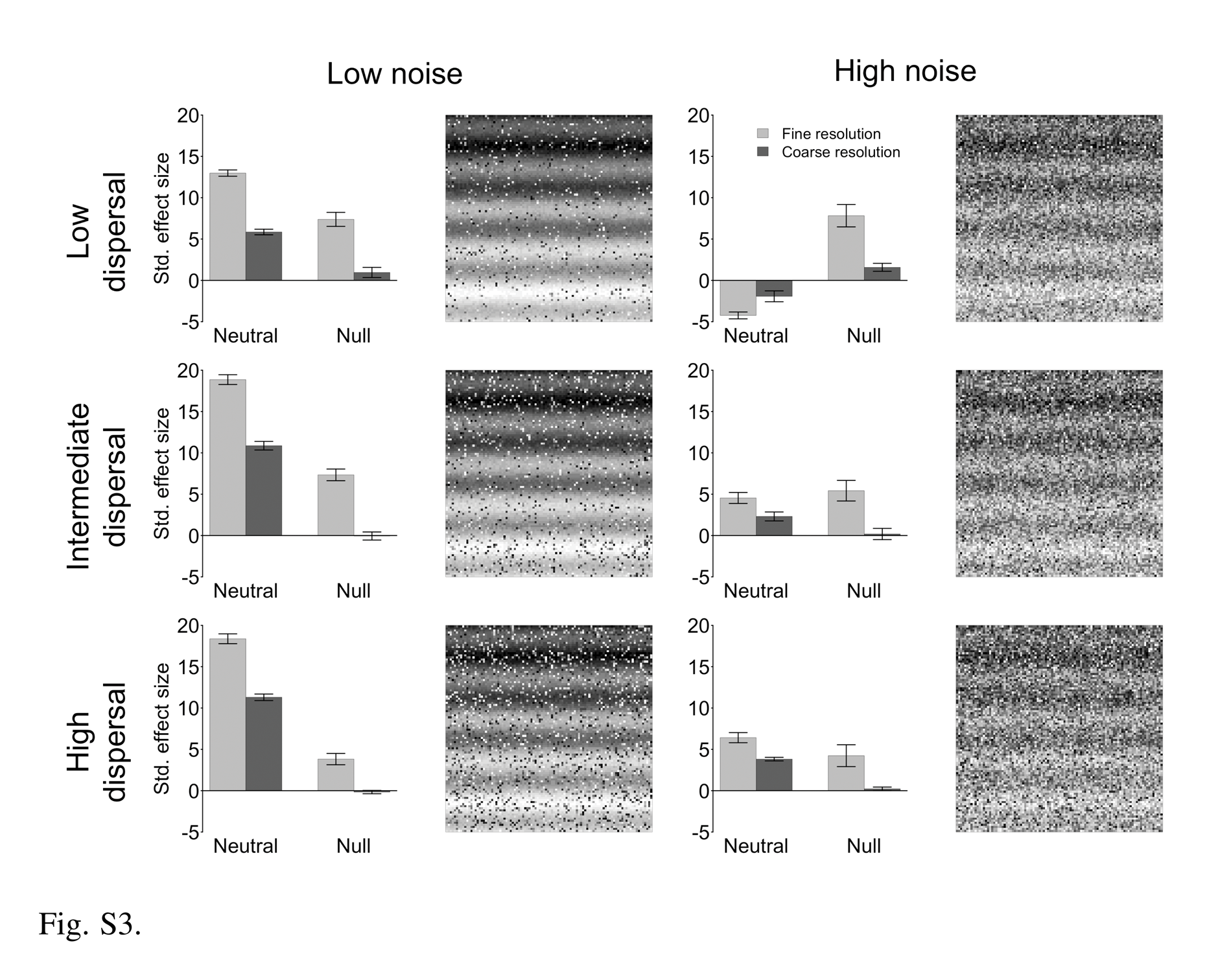

Supplement: Figure S3 — Results of the analysis performed within one of the latitudinal strata for narrow niche breadth stratified by dispersal (rows) and noise (columns). Next to each simulated community, mean (S.E.) standardised effect size are reported with data stratified by type of null hypothesis (neutral vs. null) and sampling design. (TIF) [file pone.0035942.s003.tif]

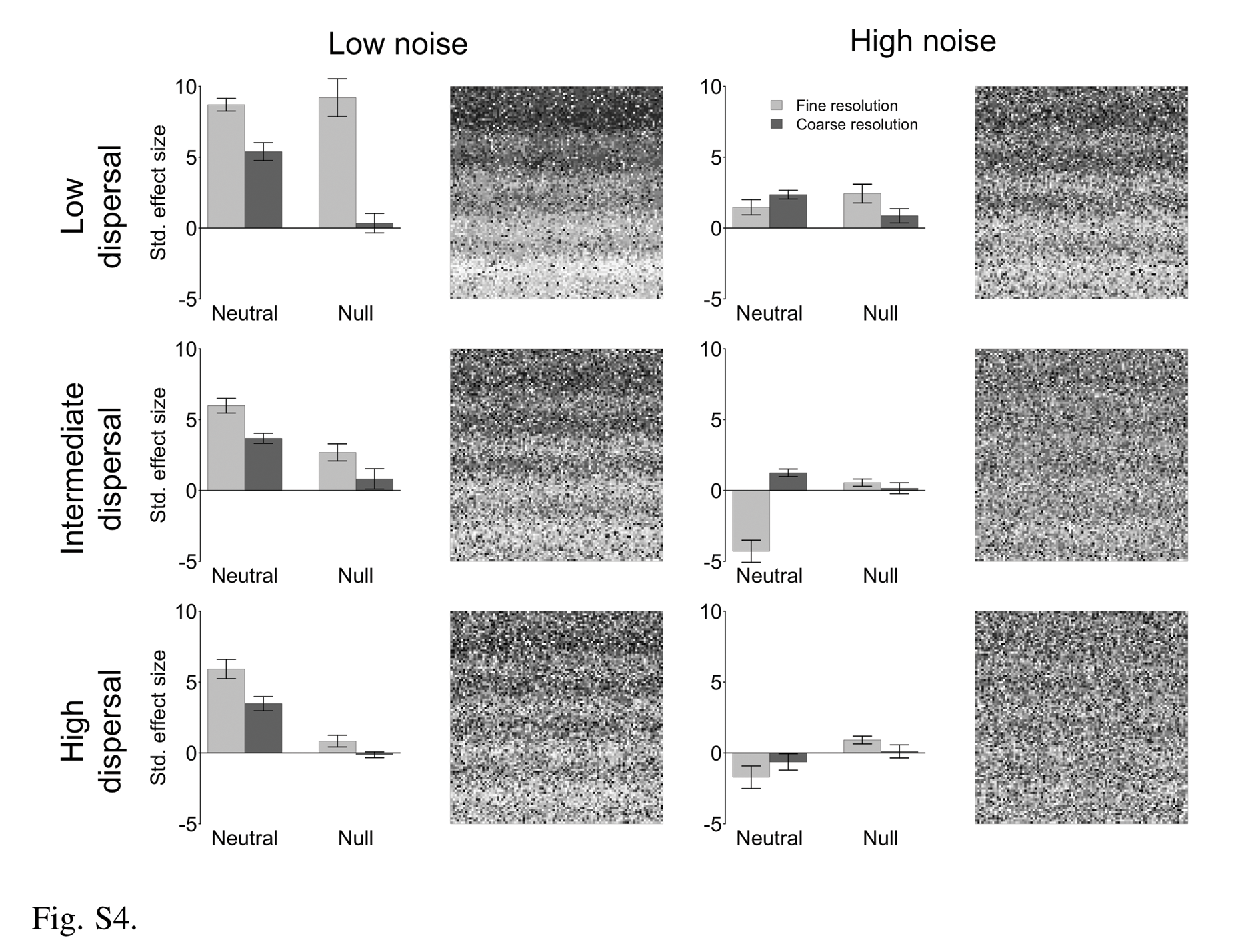

Supplement: Figure S4 — Results of the analysis performed within one of the latitudinal strata for intermediate niche breadth stratified by dispersal (rows) and noise (columns). Next to each simulated community, mean (S.E.) standardised effect size are reported with data stratified by type of null hypothesis (neutral vs. null) and sampling design. (TIF) [file pone.0035942.s004.tif]

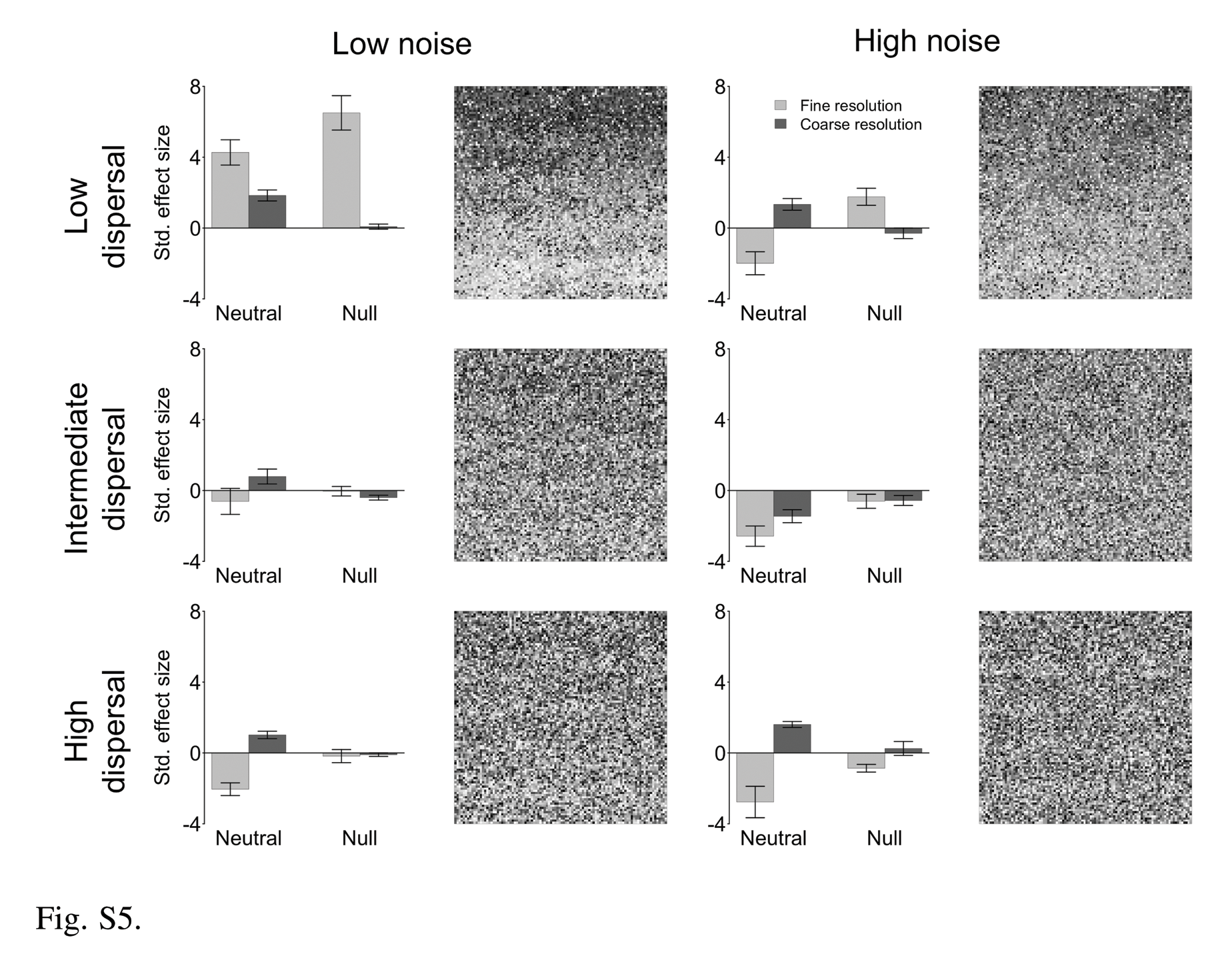

Supplement: Figure S5 — Results of the analysis performed within one of the latitudinal strata for wide niche breadth stratified by dispersal (rows) and noise (columns). Next to each simulated community, mean (S.E.) standardised effect size are reported with data stratified by type of null hypothesis (neutral vs. null) and sampling design. (TIF) [file pone.0035942.s005.tif]
