# Supplementary material for: Compositional Divergence and Convergence in Local Communities and Spatially Structured Landscapes
Source: PLoS One. 2012 Apr 26;7(4):e35942. doi: 10.1371/journal.pone.0035942 (PMC3338555; doi:10.1371/journal.pone.0035942)
Supplement: Table S1 — Comparison of scenarios based on random spatial distribution with scenarios based on a spatially structured niche axis. Values refer to average standardised effect size (mean ± S.E.) of the C-score from null model analysis. (DOC) [file pone.0035942.s006.doc]

Table S1. Comparison of scenarios based on random spatial distribution with scenarios based on a spatially structured niche axis. Values refer to average standardised effect size (mean ± S.E.) of the C-score from null model analysis.

| Fine Resolution sampling | Narrow Niche,  Low Dispersal,  Low Noise | Broad Niche,  High Dispersal,  High Noise |
| --- | --- | --- |
| Random Landscape | -0.142 ± 0.324 | -0.285 ± 0.343 |
| Spatially Structured Landscape | 17.140 ± 1.643 | -0.331 ± 0.348 |
|  |  |  |
| Coarse Resolution sampling | Narrow Niche,  Low Dispersal,  Low Noise | Broad Niche,  High Dispersal,  High Noise |
| Random Landscape | 0.850 ± 0.450 | -0.087 ± 0.220 |
| Spatially Structured Landscape | 3.542 ± 0.694 | 0.098 ± 0.439 |
