# Supplementary material for: Compositional Divergence and Convergence in Local Communities and Spatially Structured Landscapes
Source: PLoS One. 2012 Apr 26;7(4):e35942. doi: 10.1371/journal.pone.0035942 (PMC3338555; doi:10.1371/journal.pone.0035942)
Supplement: Table S2 — Comparing results from multiple sampling within one landscape with results from sampling replicated landscapes. Values refer to average standardised effect size (mean ± S.E.) of the C-score from null model analysis. (DOC) [file pone.0035942.s007.doc]

Table S2. Comparing results from multiple sampling within one landscape with results from sampling replicated landscapes. Values refer to average standardised effect size (mean ± S.E.) of the C-score from null model analysis

| Fine Resolution sampling | Narrow Niche,  Low Dispersal,  Low Noise | Broad Niche,  High Dispersal,  High Noise |
| --- | --- | --- |
| Single sample from replicated Landscapes | 16.58 ± 0.32 | -0.3 ± 0.07 |
| Multiple sampling within one Landscape | 17.14 ± 1.64 | -0.3 ± 0.35 |
|  |  |  |
| Coarse Resolution sampling | Narrow Niche,  Low Dispersal,  Low Noise | Broad Niche,  High Dispersal,  High Noise |
| Single sample from replicated Landscapes | 2.36 ± 0.65 | -0.01 ± 0.204 |
| Multiple sampling within one Landscape | 2.51 ± 0.694 | -0.05 ± 0.44 |
