# Supplementary material for: Compositional Divergence and Convergence in Local Communities and Spatially Structured Landscapes
Source: PLoS One. 2012 Apr 26;7(4):e35942. doi: 10.1371/journal.pone.0035942 (PMC3338555; doi:10.1371/journal.pone.0035942)
Supplement: Table S3 — Comparing results from multiple sampling within one landscape with results from sampling replicated landscapes. Values refer to average standardised effect size (mean ± S.E.) from neutral analysis. (DOC) [file pone.0035942.s008.doc]

Table S3. Comparing results from multiple sampling within one landscape with results from sampling replicated landscapes. Values refer to average standardised effect size (mean ± S.E.) from neutral analysis

| Fine Resolution sampling | Narrow Niche,  Low Dispersal,  Low Noise | Broad Niche,  High Dispersal,  High Noise |
| --- | --- | --- |
| Single sample from replicated Landscapes | 11.26 ± 0.17 | -1.94 ± 0.343 |
| Multiple samples within one Landscape | 11.58 ± 0.86 | -2.75 ± 1.03 |
|  |  |  |
| Coarse Resolution sampling | Narrow Niche,  Low Dispersal,  Low Noise | Broad Niche,  High Dispersal,  High Noise |
| Single sample from replicated Landscapes | 3.51 ± 0.05 | -1.31 ± 0.13 |
| Multiple samples within one Landscape | 4.54 ± 0.69 | -2.10 ± 0.42 |
