# Supplementary material for: Compositional Divergence and Convergence in Local Communities and Spatially Structured Landscapes
Source: PLoS One. 2012 Apr 26;7(4):e35942. doi: 10.1371/journal.pone.0035942 (PMC3338555; doi:10.1371/journal.pone.0035942)
Supplement: Table S4 — ANOVA table for the linear model with Standardised Effect Size (within Latitudinal zones; see methods for details) as response. Factors were Method of analysis (neutral, null), Sampling design (Fine, Coarse), Niche Breadth (narrow, medium, broad), Dispersal (low, intermediate, high) and noise (low and high). This table shows overall main and interaction (:) effects. (DOC) [file pone.0035942.s009.doc]

Table S4. ANOVA table for the linear model with Standardised Effect Size (**within** Latitudinal zones; see methods for details) as response. Factors were Method of analysis (neutral, null), Sampling design (Fine, Coarse), Niche Breadth (narrow, medium, broad), Dispersal (low, intermediate, high) and noise (low and high). This table shows overall main and interaction (:) effects.

| Effect | Df | Sum Sq | Mean Sq | F value | Pr(>F) |
| --- | --- | --- | --- | --- | --- |
| Method (Neutral vs Null) | 1 | 272 | 272 | 41 | < 0.001 |
| Resolution (Fine vs Coarse) | 1 | 406 | 406 | 61 | < 0.001 |
| Niche Breadth (Narrow, Medium, Broad) | 2 | 1933 | 966 | 145 | < 0.001 |
| Disperal (Low, Intermediate, High) | 2 | 70 | 35 | 5 | < 0.001 |
| Noise (Low vs High) | 1 | 1362 | 1362 | 204 | < 0.001 |
| Method:Niche Breadth | 2 | 414 | 207 | 31 | < 0.001 |
| Method:Resolution | 1 | 140 | 140 | 21 | < 0.001 |
| Student:Niche Breadth | 2 | 495 | 248 | 37 | < 0.001 |
| Niche Breadth:Dispersal | 4 | 481 | 120 | 18 | < 0.001 |
| Resolution:Noise | 1 | 291 | 291 | 44 | < 0.001 |
| Method:Noise | 1 | 807 | 807 | 121 | < 0.001 |
| Residuals | 413 | 2761 | 7 |  |  |

Df, degrees of freedom; Sum Sq, sum of squares; Mean Sq, mean sum of squares.
