# Supplementary material for: Compositional Divergence and Convergence in Local Communities and Spatially Structured Landscapes
Source: PLoS One. 2012 Apr 26;7(4):e35942. doi: 10.1371/journal.pone.0035942 (PMC3338555; doi:10.1371/journal.pone.0035942)
Supplement: Text S1 — The theoretical basis of the work is briefly recalled. (DOC) [file pone.0035942.s010.doc]

Supporting Information

Supplementary methods for the simulation framework

Theoretical background:

Competition can be modelled in a stochastic way if one assumed that the survival of a propagule would be best expressed by some probability function, which should depend on the value *E* of the niche axis the propagule experiences locally. A good choice is the normal distribution (Tilman 2004; Gravel et al. 2006), for which the survival probability *s_i_(E)* of species *i* is

eq. 1

where *o_i_* is the optimal value of the environmental axis for species *i*, i.e. the value at which the species is the best competitor. Sigma (σ_n_) is the variance in the optimal environmental value and an optimal quantitative representation of the concept of niche breadth. Indeed, imagining each individual as a cell in a flat landscape of cells (Individual Based Model), dispersal can be introduced through expressing the probability to recruit a given species after a cell died as a function of both survival probability and dispersal from the neighbourhoods of the dead cell, i.e.

eq.2

where *W* is a weighting matrix accounting for the dispersal kernel and *d* is the distance separating parents and propagules. Dispersal events over long distances are less probable, and the probability of recruitment strongly decreases if the propagule falls far from the niche optimum. This spatially explicit model thus describes the process of recruitment in terms of stochastic niche dynamics and dispersal, allowing control over the degree of neutrality by varying niche overlap and dispersal rates.
